# Phosphoinositide 3-Kinase C2β Regulates RhoA and the Actin Cytoskeleton through an Interaction with Dbl

**DOI:** 10.1371/journal.pone.0044945

**Published:** 2012-09-12

**Authors:** Karolina Błajecka, Marin Marinov, Laura Leitner, Kristin Uth, Guido Posern, Alexandre Arcaro

**Affiliations:** 1 Department of Clinical Research, University of Bern, Bern, Switzerland; 2 Department of Oncology, University Children’s Hospital Zurich, Zurich, Switzerland; 3 Department of Molecular Biology, AG Regulation of Gene Expression, Max-Planck-Institute of Biochemistry, Martinsried, Germany; Karolinska Institutet, Sweden

## Abstract

The regulation of cell morphology is a dynamic process under the control of multiple protein complexes acting in a coordinated manner. Phosphoinositide 3-kinases (PI3K) and their lipid products are widely involved in cytoskeletal regulation by interacting with proteins regulating RhoGTPases. Class II PI3K isoforms have been implicated in the regulation of the actin cytoskeleton, although their exact role and mechanism of action remain to be established. In this report, we have identified Dbl, a Rho family guanine nucleotide exchange factor (RhoGEF) as an interaction partner of PI3KC2β. Dbl was co-immunoprecipitated with PI3KC2β in NIH3T3 cells and cancer cell lines. Over-expression of Class II phosphoinositide 3-kinase PI3KC2β in NIH3T3 fibroblasts led to increased stress fibres formation and cell spreading. Accordingly, we found high basal RhoA activity and increased serum response factor (SRF) activation downstream of RhoA upon serum stimulation. In contrast, the dominant-negative form of PI3KC2β strongly reduced cell spreading and stress fibres formation, as well as SRF response. Platelet-derived growth factor (PDGF) stimulation of wild-type PI3KC2β over-expressing NIH3T3 cells strongly increased Rac and c-Jun N-terminal kinase (JNK) activation, but failed to show similar effect in the cells with the dominant-negative enzyme. Interestingly, epidermal growth factor (EGF) and PDGF stimulation led to increased extracellular signal-regulated kinase (Erk) and Akt pathway activation in cells with elevated wild-type PI3KC2β expression. Furthermore, increased expression of PI3KC2β protected NIH3T3 from detachment-dependent death (anoikis) in a RhoA-dependent manner. Taken together, these findings suggest that PI3KC2β modulates the cell morphology and survival through a specific interaction with Dbl and the activation of RhoA.

## Introduction

The phosphoinositide-3 kinase (PI3K) family comprises evolutionary conserved lipid kinases characterised by their ability to phosphorylate phosphoinositides at the D-3 position of the inositol ring. This reaction leads to the production of essential for the cell second messengers as PI(3)P, PI(3,4)P_2_ and PI(3,4,5)P_3_ [1]. The subsequent membrane recruitment of enzymes recognizing and binding D-3 phosphorylated phosphoinositides leads to their activation, which results in the initiation of multiple signalling cascades providing growth, survival and motility signals to the cell. The biological functions of PI3K have been widely investigated by the use of transgenic mouse models and *in vitro* cell systems. It became clear that PI3K are extremely important for cell proliferation, differentiation, chemotaxis and motility. Furthermore, they are implicated in the embryonic development, immunity and the development of diseases such as cancer, inflammation and diabetes [1]–[3]. The family of PI3K comprises three classes, based on structural similarity and *in vitro* substrate specificity. Class I PI3K is divided into Class IA, comprising three isoforms activated by growth factor receptor tyrosine kinases (RTKs) and Class IB with a single member activated by the Gβγsubunit downstream of activated G-protein coupled receptors (GPCR) [1]. The main product of Class I PI3K activity, the lipid second messenger PI(3,4,5)P_3_, activates a plethora of downstream molecules by binding to their pleckstrin-homology (PH) domains [4]. To one of the best studied effectors belong the serine/threonine kinase Akt [5] and RhoGEFs [6].

Class II PI3K consists of three members termed PI3KC2α, PI3KC2β and PI3KC2γ. *In vitro*, the main substrates of these enzymes are PI and PI(4)P [7], [8]. Class II PI3K are activated downstream of RTK (EGFR, PDGFR and Kit), cytokine receptors, insulin and integrins [9]–[13]. Localisation of PI3KC2α in the trans-Golgi apparatus and in clathrin vesicles indicates function in clathrin assembly and clathrin mediated trafficking [14], [15]. Further studies have revealed that PI3KC2β is implicated in the cytoskeletal regulation and migration of cancer cells [16]–[18]. However, the specific functions of Class II PI3K in response to their activators remain to be fully understood.

The control of actin filaments dynamic is crucial for cell morphology, adhesion and movement [19]. PI3Ks regulate cell polarity and motility by controlling multiple proteins and protein complexes implicated in cytoskeletal rearrangement and actin dynamics [20]. It was recently demonstrated in *Drosophila melanogaster* hemocytes that Pi3K68D in collaboration with endosomal myotubularin PI3K phosphatase Mtm-1 coregulates the PI(3)P pool, which in turn promotes F-actin reorganization and modulates protrusion formation [21]. *D. melanogaster* Pi3K68D is a homologue of human PI3KC2β and human *Mtm* genes present high functional conservation with the *D. melanogaster* MTM family members, which suggests that the MTM/Class II PI3K pathway might be important for similar biological responses in mammalian cells [21]. However, the main players in the regulation of actin dynamics are GTP-binding proteins known as RhoGTPases. A recent report in vascular smooth muscle cells of spontaneously hypertensive rats showed that Class II PI3KC2α is involved in Ca^2+^-induced Rho signaling pathway activation, which stimulates the interaction of myosin II (MII) with actin filaments, which in turn leads to increased cell contractility [22]. RhoGTPases proteins cycle between inactive GDP- and active GTP-bound states and hydrolyse GTP to GDP. This process is tightly regulated by guanine nucleotide exchange factors (GEFs), GTPase-activating proteins (GAPs) and guanine nucleotide dissociation inhibitors (GDIs). The best studied Rho GTPases under the control of PI3K activity are RhoA, Rac1 and Cdc42 [23]. Rac1 is involved in the formation of membrane extensions (lamellipodia or ruffles) along the leading edge of the cell. Cdc42 controls the formation of spike-like membrane extensions (filopodia), involved in the recognition of the extracellular environment. RhoA stabilises the cell body and promotes cell attachment to the extracellular matrix by inducing the formation of stress fibres and focal adhesions [24]. Ligand binding to RTK, integrins and GPCR results in PI3K activation and accumulation of PI(3,4)P_2_ and PI(3,4,5)P_3_ in the cell membrane. This process is spatially defined by the site of ligand concentration and results in the local membrane recruitment of GEFs [25]. The *dbl* oncogene was the first GEF identified by cloning from human diffuse B-cell lymphoma and subsequently from nodular poorly differentiated lymphoma (NPDL), which upon transfection into NIH3T3 cells induced cellular transformation [26], [27]. The oncogenic form of *dbl* arises from full length *dbl* as a result of amino acid truncation of its N-terminus, which is crucial for the increased transforming activity of *dbl* oncogene [28], [29]. The mechanism of proto-Dbl regulation is strongly dependent on its multi-domain structure. As many other GEFs family members, it contains Dbl homology-Pleckstrin homology (DH-PH) tandem domains. These domains are respectively responsible for the intrinsic GEF catalytic activity toward RhoGTPases and the intracellular distribution to the plasma membrane or to the cytoskeletal matrix [30]–[32]. The spectrin domain of proto-Dbl located at the N-terminus regulates Dbl activity by indirect binding to its PH-domain, thus blocking the access to the catalytic DH domain and masking the intracellular targeting function of the PH domain [31]–[33]. Many GEFs exist in an inactive or partially active state maintained by complex intra- and intermolecular interactions. These inhibitory conformations can be altered by upstream regulatory signals, which involve phosphorylation, oligomerization, interactions with heterotrimeric G-protein subunits, protein kinases, adaptor or scaffolding proteins, or phosphoinositol lipids, and result in intracellular translocation and stimulation of the GEF catalytic activity [34]–[36]. Once GEFs are at the membrane, they assemble into multi-protein complexes with PI3K, RhoGTPases and actin-binding proteins [37]. These multi-protein complexes are crucial for the proper localisation to dynamic actin structures and the efficient activation of RhoGTPases [37], [38].

In our previous study, we reported the implication of PI3KC2β in a multi-protein complex regulating Rac activity in A-431 epithelial carcinoma cells [17]. In order to investigate the function of PI3KC2β in non-tumor cells, we established clones of NIH3T3 fibroblast stably expressing either the wild-type or dominant-negative form of the enzyme. According to our previous data, we found elevated Rac/JNK activation upon PDGF stimulation in PI3KC2β wild-type expressing cells. We further observed increased cell spreading and strong stress fibres formation due to elevated RhoA activity. We show that serum stimulation leads to strong serum response factor (SRF) induction downstream of RhoA and polymerised actin. This finding led us to the identification of a RhoGEF Dbl as interaction partner of PI3KC2β. Hence, we hypothesise that in NIH3T3 cells PI3KC2β is involved in a complex comprising Dbl and regulates RhoA and Rac1 activity. This signalling cascade further protected the cells from detachment-induced apoptosis.

## Materials and Methods

### Reagents and Antibodies

The following antibodies were used: PI3KC2β was described in [7], 9E10 myc epitope, p115RhoGEF, Dbl, Caveolin-2, Grb2, Src, RhoA, GFP, p53 (Santa Cruz Biotechnology, Santa Cruz, CA, USA); Caveolin-1, Akt (pan), phospho-Akt (Ser473), phospho-Akt (Thr308), phospho-SAPK/JNK (Thr183/Tyr185), phospho-S6 ribosomal protein (Ser240/244), phospho-GSK-3α/β (Ser21/9), phospho-FOXO1 (Thr24)/FOXO3a (Thr32), phospho-Erk1/2 (Thr202/Tyr204), p44/42 MAPK, Bcl-2, Bcl-XL, PTEN, phospho-Bad (Ser136) (Cell Signalling Technology, Danvers, MA, USA); FITC-conjugated anti-mouse and anti-rabbit antibodies, β-actin, anti-mouse IgG (Sigma-Aldrich, St Louis, MO, USA); anti-rabbit-IgG (R&d Systems, Minneapolis, MN,USA); Rac1 (BD Transduction Laboratories, USA). Anti-EE antibody was kindly provided by Julian Downward (CRUK). Alexa Fluor 555 was obtained from Invitrogen. Recombinant EGF, PDGF BB were purchased from Calbiochem, La Jolla,CA,USA. Oleoyl-L-α-lysophosphatidic acid (LPA) was purchased from Sigmla-Aldrich, St Louis, MO, USA.

The SRF luciferase reporter plasmid p3DA-Luc has been described previously [39].

### Cell Culture

Mouse NIH3T3 fibroblast, HEK293 and COS-1 cells were purchased from the American Type Culture Collection. Mouse NIH3T3 fibroblast, HEK293 and COS-1 cells were grown in DMEM (Life Technologies/Invitrogen) supplemented with 10% (v/v) FCS and penicillin/streptomycin/L-glutamine, and passaged every 3 to 5 days by trypsinization. Cultures were incubated in a humidified atmosphere of 5% CO_2_ at 37°C. Stably transfected NIH3T3 clones were grown in the presence of 0.8 mg/ml G418.

### Retroviral Infection

NIH3T3 cell lines stably expressing Glu-tagged PI3KC2β wild-type (WT) were described [9]. The cDNA construct encoding myc-tagged dominant-negative kinase-dead PI3K-C2β (DN) [17] was subcloned into the pBabe Neo Vector. Retroviral packaging BOSC 23 cells were transfected using a Ca^2+^ phosphate protocol. NIH3T3 cells were grown up to 40–60% confluence and infected with the recombinant retrovirus for 48 h at 37°C. The cultures were split 1∶10 in DMEM- 10% FCS and 0.8 mg/ml G418 containing medium. Colonies of resistant cells appeared after 1 week. Single colonies were picked and expanded in selective medium. Protein expression was confirmed by western blot analysis. All experiments were done with at least two clones from each transfection. Cells transduced with pBabe Neo vector without insert were used as controls.

**Figure 1:**
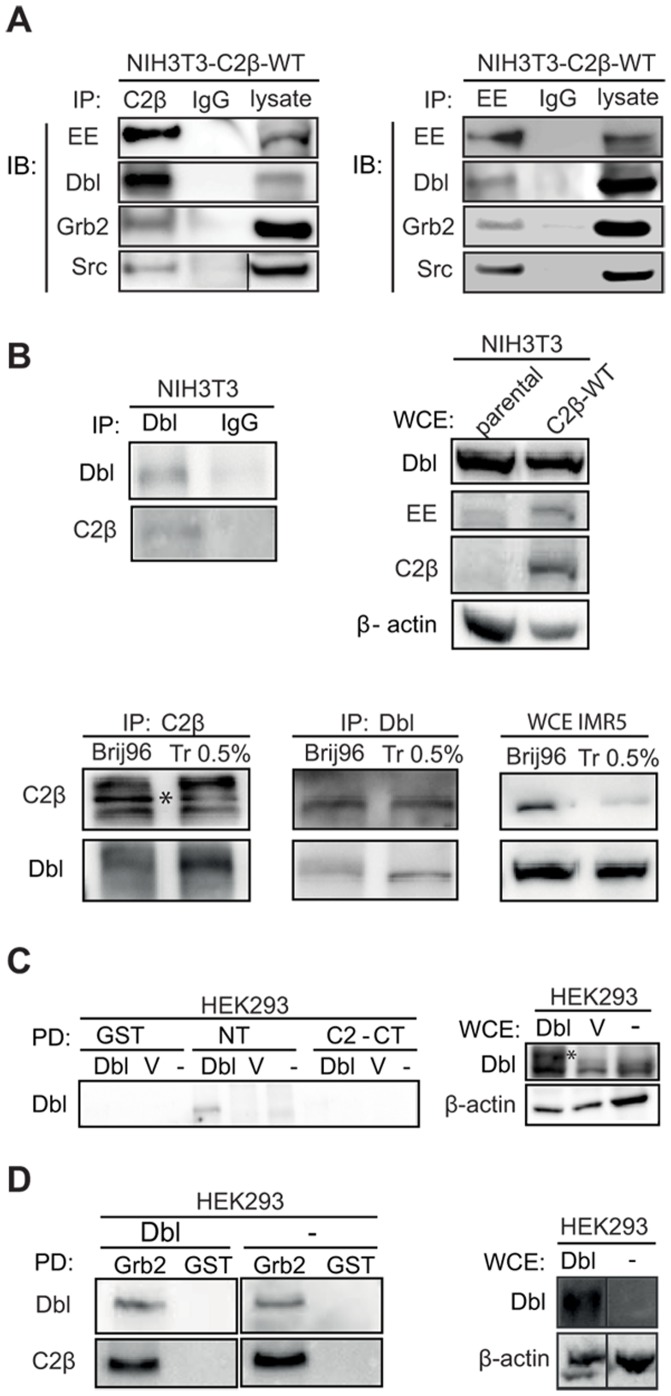
PI3KC2β interacts with Dbl GEF for Rho GTPases. (A) Lysates of NIH3T3 cells stably overexpressing wild-type Glu-PI3KC2β (NIH3T3-C2β-WT) were immunoprecipitated (IP) with an anti-C2β, anti-Glu (EE) tag, anti-Dbl or control IgG antibody. PI3KC2β interaction with the endogenous Dbl, as well as Grb2 and Src was detected by immunoblotting (IB) with the indicated antibodies. (B) Complex of endogenous PI3KC2β and Dbl was immunoprecipitated from the whole cell extract (WCE) of the parental NIH3T3 cells and IMR5 neuroblastoma human cancer cells with anti-Dbl, anti-C2β and control IgG antibody. Samples were subjected to western blot analysis with the indicated antibodies. In the case of IMR5 cells Triton 0.5% and Brij96 1% lysis buffers were used, while NIH3T3 cells were lysed with Triton 1%. Asterisk indicates PI3KC2β. (C) Interaction between the PI3KC2β and Dbl was examined by the pull-down (PD) of PI3KC2β GST-fused N-terminal and C2 C-terminal domains in HEK293 cells transfected with HA-proto-Dbl (Dbl), pcDNA3 empty vector (V) or untransfected control (-). Complex formation was shown by immunoblotting with anti-Dbl antibody. Asterisk indicates ectopically expressed (HA-tag) Dbl. (D) Interaction between the Dbl and Grb2 was examined by the GST-Grb2 pull-down in HA-proto-Dbl (Dbl) transfected and untransfected (-) HEK293 cells. Pull-down samples were subjected to western blot analysis with indicated antibodies. All experiments were repeated at least two times with similar results.

### Transient Transfections

HEK293 cells were transiently transfected with a Ca^2+^ phosphate protocol [7], while NIH3T3 and COS-1 cells were transfected with Lipofectamine or Lipofectamine 2000 (Invitrogen) respectively, according to the manufacturer’s instructions.

### Growth Factor Stimulations

To investigate Erk and Akt signalling downstream of EGFR and PDGFR in NIH3T3 cells stably expressing PI3KC2β wild-type (WT) and dominant-negative (DN) were grown to confluency in a six-well plate and starved overnight in DMEM containing 0.5% FCS and penicillin/streptomycin/L-glutamine. Cells were then stimulated with the EGF (1 nM) and PDGF (1 nM) for 10 min. at 37°C, as indicated in the figure legends. Cellular lysates were prepared as described below.

Stimulation of cells with PDGF and LPA for investigation of RhoA and Rac1 activity is described below in the section of RhoGTPases activation assay.

**Figure 2:**
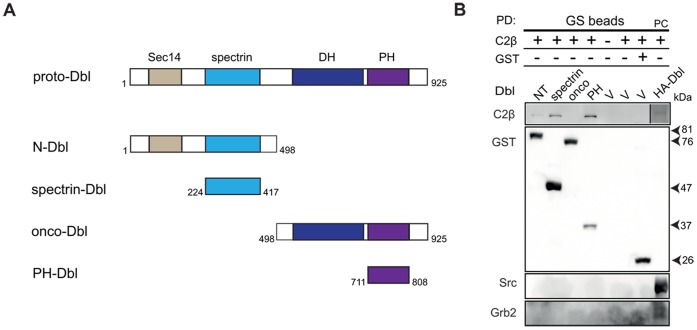
PI3KC2β binds with high affinity to the spectrin- and PH- Dbl domain. (A) Schematic representation of Dbl mutant constructs, which were kind gift of Prof. Danny Manor from the School of Medicine at Case Western Reserve University, Cleveland, USA. (B) COS-1 cells co-transfected with Myc-PI3KC2β and different GST-fused Dbl domains (N-, spectrin-, onco-, PH-) in pEBG vector were subjected to gluthatione-sepharose beads affinity-purification and immunobotted with indicated antibodies. Experiment was repeated at least two times with similar results. PC – positive control; COS-1 cells co-transfected with PI3KC2β and HA-proto-Dbl, and immunoprecipitated with anti-Dbl antibody.

### SDS/PAGE and Western-blot Analysis

NIH3T3-C2β-WT and –DN cellular lysates were prepared with RIPA buffer (50 mM Tris.Cl pH 7.4, 150 mM NaCl, 1 mM EDTA, 1% Triton X-100, 1% sodium deoxycholate, 0.1% SDS, 10% glycerol, 10 µM leupeptin, 10 µM pepstatin A, 1 mM phenylmethylsulfonylfluorid [PMSF], 5 mM iodoacetamide, 5 mM benzamidine, 2 mM sodium orthovanadate [http://en.wikipedia.org/wiki/Chemical_formulaNa3VO4] and 10 mM sodium fluoride [NaF]). In the case of NIH3T3, COS1 and HEK293 cells lysis buffer was changed to Triton 1% (50 mM Tris.Cl pH 7.4, 150 mM NaCl, 1 mM EDTA, 0.5 mM EGTA, 1% Triton X-100, 0.5% NP-40) supplemented with 7×concentrated stock of protease inhibitors (Complete Mini, Roche) and phosphatase inhibitors (1 mM NaF, 1 mM Na_3_VO_4_, 10 mM β-glycerophosphate). For the studies of endogenous complex formation in IMR5 cells Triton 0.5% and Brij96 1% (50 mM Tris.Cl pH 7.4, 150 mM NaCl, 1 mM EDTA, 0.5 mM EGTA, 1% Brij96) lysis buffers supplemented with 7×concentrated stock of protease inhibitors (Complete Mini, Roche) and phosphatase inhibitors (1 mM NaF, 1 mM Na_3_VO_4_, 10 mM β-glycerophosphate) were used, as indicated in the figure legends. Prior to lysis cells were washed once with ice-cold phosphate-buffered saline (PBS), lysed on ice for 20 min., and centrifuged at 16,000×*g* for 25 min. at 4°C. The supernatants were collected and normalized for protein content by using the BCA (bicinchoninic acid) protein assay kit (Pierce). 2x or 5x SDS sample buffer (50 mM Tris.HCl, pH 6.8, 2%/5% SDS, 10% glycerol, 200 mM DTT, and 0.25% bromophenol blue) was then added to the samples, followed by denaturation for 3 min. at 100°C and analysed by SDS/PAGE. The gels were transferred to a hydrophobic PVDF membrane (Hybond-P; Amersham Biosciences) by electrophoresis. The membranes were then blocked in 1× PBS, 5% (w/v) BSA (phospho-specific antibodies) or 1× PBS/3% non-fat dry milk (all other antibodies) for 16 h at 4°C. The membranes were incubated with primary antibodies (diluted according to the manufacturer’s protocol) for 16 h at 4°C. After washing in 1× PBS and 0.1% (w/v) Tween 20, the immunoblots were incubated with donkey anti-rabbit IgG or sheep anti-mouse IgG secondary antibodies (1∶10000 dilution) coupled with horseradish peroxidase (Amersham Biosciences) for 1 h at room temperature. After washing of the immunoblots, chemiluminescence was used for detection, using either the ECL® (enhanced chemiluminescence) western blotting detection reagents (Amersham Biosciences) or SuperSignal West Femto Maximum Sensitivity Substrate (PIERCE) according to the manufacturer’s protocol.

**Figure 3:**
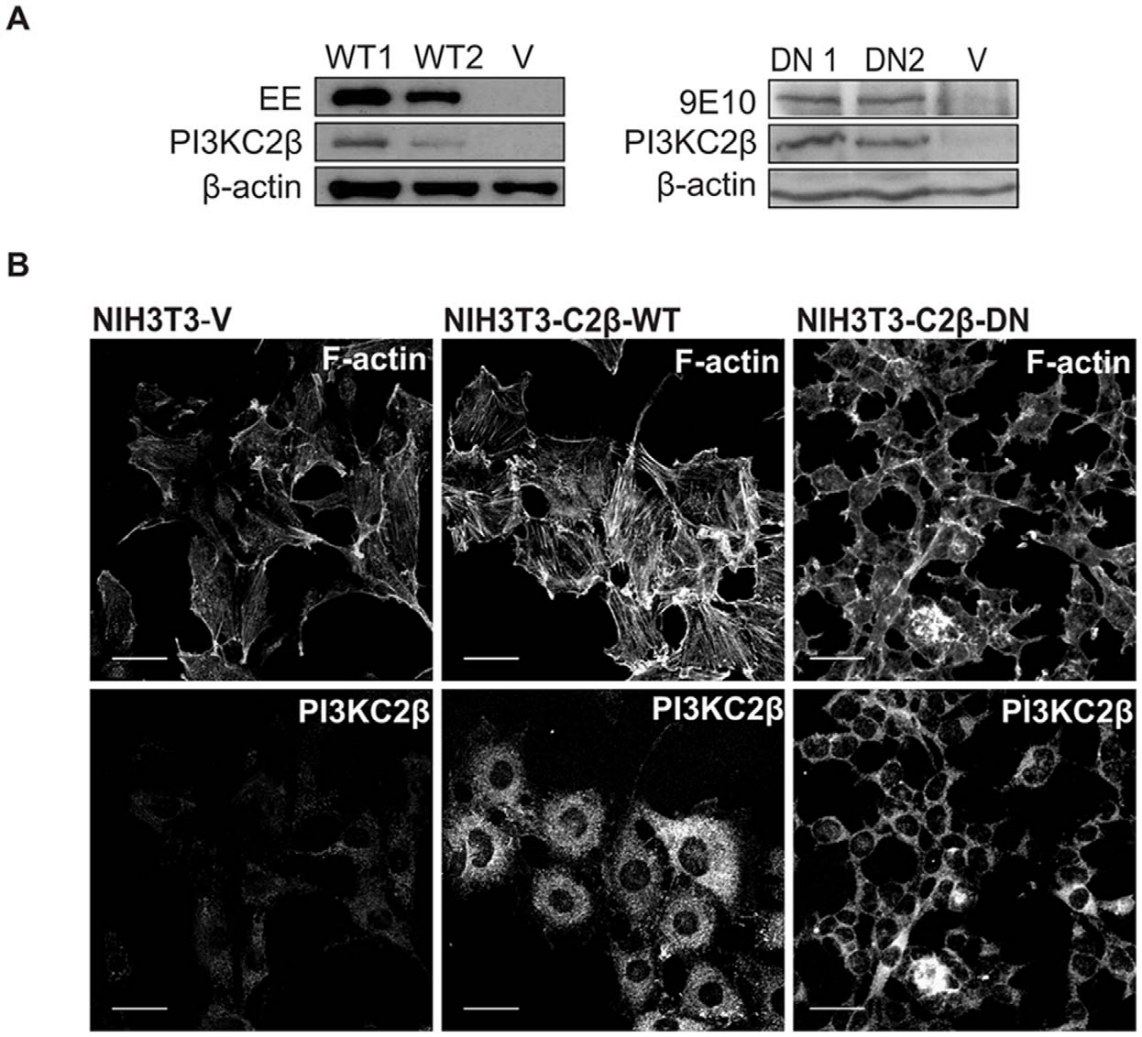
Kinase-dependent PI3KC2β effect on cytoskeletal rearrangements. (A) Cell lysates of NIH3T3 cells stably expressing empty vector (NIH3T3-V), kinase-dead (D1213A) dominant-negative Myc-PI3KC2β (NIH3T3-C2β-DN) and wild-type Glu-PI3KC2β (NIH3T3-C2β-WT) were analysed for the recombinant PI3KC2β expression by immunoblotting with the indicated antibodies (EE: Glu-tag, 9E10: Myc-tag). (B) Confocal images of NIH3T3-V, -C2β-WT and –C2β-DN cells grown on cover slips for 24 h in 10% FCS and stained with Alexa Fluor 555 dye to localise F-actin, and an anti-PI3KC2β antibody follwed by FITC-labelled anti-rabbit antibody, to localize the kinase. WT1, WT2 and DN1, DN2 indicate individual clones. Scale bar represents 40 µm.

### Immunoprecipitations

NIH3T3-C2β-WT, NIH3T3 parental and IMR5 cells were grown to confluence in 10-cm dishes, washed with ice cold phosphate-buffered saline (PBS) before lysis on ice for 20 min. in 0.5–1 ml of lysis buffer (as indicated in the previous section). Insoluble material was removed by centrifugation for 30 min. at 16,000×*g* in 4°C. Immunoprecipitation was performed for 2 h at 4°C with primary antibodies (diluted according to the manufacturer’s instructions). IP Matrix (ExactaCruz, Santa Cruz Biotechnology) or Protein A- or G-Sepharose 4 Fast Flow beads (GE Healtcare) were then added, and the incubation was continued for 1 h at 4°C. The immunoprecipitates were washed three times in the lysis buffer and resuspended in 2x SDS sample buffer (50 mM Tris.HCl, pH 6.8, 2% SDS, 10% glycerol, 200 mM DTT, and 0.25% bromophenol blue). Samples were denatured for 3 min. at 100°C and analysed by SDS/PAGE and western blot.

**Figure 4:**
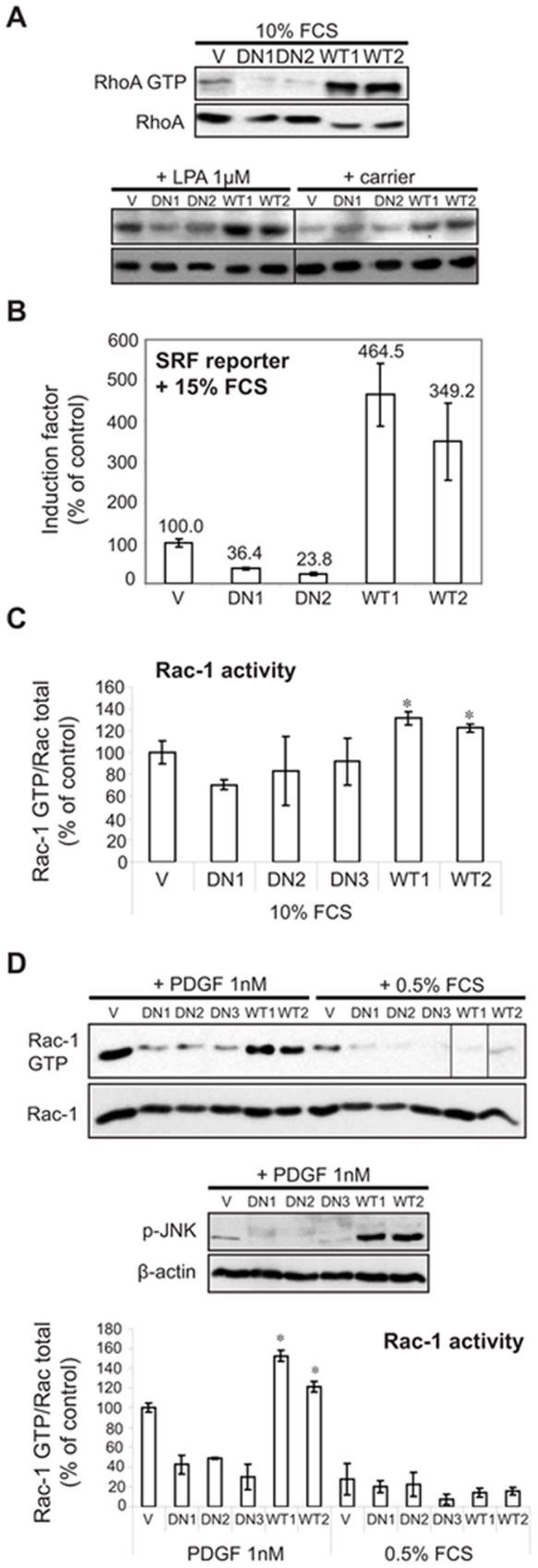
PI3KC2β regulates RhoA and Rac1 activity. (A) PI3KC2β up-regulates RhoA activity. NIH3T3-V, -C2β-DN and –C2β–WT cells were grown in 10% FCS or serum-starved for 24 h and stimulated with 1 µM LPA for 2 min. Cell lysates were equalized for protein content and subjected to GST-rhotekin pull-down as measure of RhoA activity. GTP-bound and total RhoA were detected with an anti-RhoA antibody. (B) PI3KC2β mediates RhoA-dependent serum response factor (SRF) activation. NIH3T3-V, -C2β-DN and -C2β-WT cells were transiently transfected with the SRF luciferase reporter plasmid p3DA-Luc and pRL-TK control construct. Cells were maintained in 0.5% FCS for 24 h prior to stimulation with 15% FCS for 7 h. Luciferase activity was normalized to pRL-TK control. Figure shows mean ± SD of three independent experiments. (C) PI3KC2β up-regulates Rac1 activity. NIH3T3-V, -C2β-DN and -C2β-WT cells were grown in 10% FCS. Cell lysates were equalized for protein content and subjected to GST-PAK CRIB pull-down as measure of Rac-1 activity. GTP-bound and total Rac1 were detected with anti-Rac1 antibody. Densitometry was performed and signal intensities were normalized against total Rac1. Data are mean with SD from two independent experiments. *, p<0.05. (D) PI3KC2β mediates Rac1-dependent platelet derived growth factor (PDGF) activation. NIH3T3-V, -C2β-DN and -C2β-WT cells were serum-starved for 24 h and stimulated with 1 nM PDGF for 2 min. Cell lysates were equalized for protein content and subjected to GST-PAK CRIB pull-down as measure of Rac-1 activity. GTP-bound and total Rac1 were detected with anti-Rac1 antibody. Densitometry was performed and signal intensities were normalized against total Rac1. Data are mean with SD from two independent experiments. *, p<0.05. A representative western blot is shown. Activation of JNK was assessed in the cell lines treated with PDGF by immunoblotting with phospho-specific SAPK/JNK antibody. WT1, WT2 and DN1-3 indicate individual clones.

### Production of GST-Fusion Proteins

pGEX-2T expression vectors encoding GST-PAK-CRIB, GST-rhotekin, GST-RhoA, GST-Grb2, GST-PI3KC2β N-terminal and C2 C-terminal domains were transformed into *Escherichia coli* BL21 cells. The incubation proceeded at 37°C until OD_600_ reached 0.5. Protein expression was induced with 0.2 mM isopropyl-β-D-thiogalactopyranoside (IPTG) and incubated at 30°C for 3–4 h. The bacteria were collected by centrifugation and resuspended in lysis buffer (50 mM Tris-HCl pH 7.5, 1% Triton X-100, 150 mM NaCl, 5 mM MgCl_2_, 1 mM DTT, 10 µM leupeptin, 10 µM pepstatin and 1 mM PMSF). In the case of GST-Grb2, GST-PI3KC2β N-terminal and C2 C-terminal domains lysis buffer was changed to: 50 mM Tris HCl pH 7.5, 150 mM NaCl, 5 mM EDTA pH 8.0, 1% Triton supplemented with 7×concentrated stock of protease inhibitors (Complete Mini, Roche), 1 mM PMSF, 10 µM leupeptin, 10 µM pepstatin, 5 mM benzamidin, 1 mM DTT, 1 µl/ml aprotinin, 1 mM NaF and 1 mM Na_3_VO_4_. Bacteria were thereafter sonicated on ice six times for 10 s or 3 times for 45 sec with 1 min. intervals (in the case of GST-Grb2, GST-PI3KC2β N-terminal and C2 C-terminal domains). The lysed cells were clarified by centrifugation at 30,000×*g* for 30 min. at 4°C. GST-fusion proteins were affinity-purified from the resulting supernatants by addition of glutathione-sepharose beads (Amersham Biosciences) followed by incubation at 4°C for 60 min. The glutathione-sepharose beads were collected by centrifugation at 805×*g* at 4°C followed by four washes with washing buffer (50 mM Tris-HCl pH 7.5, 0.5% Triton X-100, 150 mM NaCl, 5 mM MgCl_2_, 1 mM DTT, 10 µM leupeptin, and 0.1 mM PMSF. After the final wash, the beads containing the GST-fusion proteins were resuspended in the washing buffer supplemented with 10% glycerol. The purity of the GST-fusion proteins was assayed by SDS-PAGE.

GST-tagged NT-, spectrin-, onco- and PH- Dbl mutant constructs, described previously in [31], were expressed in COS-1 cells, lysed with HEPES buffer as in [31] and purified with glutathione-sepharose beads (GE Healthcare). Beads containing the GST-fusion proteins were washed 3–4×with ice-cold HEPES buffer and 2x with washing buffer (50 mM Tris pH 7.5, 150 mM NaCl). Next, they were resuspended in the washing buffer supplemented with 1 mM DTT and 50% glycerol and stored in −20°C.

**Figure 5:**
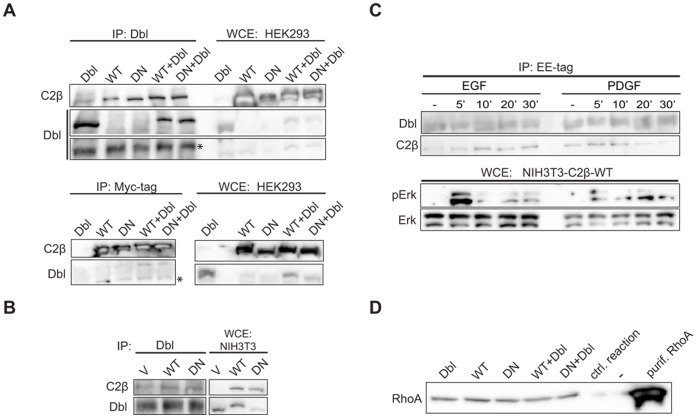
The assembly of the Dbl - PI3KC2β complex is not modulated by PI3K activity or cell stimulation with EGF or PDGF. (A) HEK293 cells were transfected with vectors encoding Dbl in combination with Myc-PI3KC2β WT or DN, or empty vector. Immunoprecipitates prepared with anti-Dbl or anti-Myc tag antibodies were analysed by western blot with the antibodies indicated. (B) Lysates from NIH3T3-V, -C2β-WT or -C2β-DN cells were immunoprecipitated with anti-Dbl antibodies and analysed by western blot. (C) Serum-starved NIH3T3-C2β-WT cells were stimulated with EGF (20 ng/ml) or PDGF (20 ng/ml) for the indicated lengths of time. Immunoprecipitates prepared with anti-Glu (EE) tag antibodies were analysed by western blot with the antibodies indicated. (D) HEK293 cells were transfected with vectors encoding Dbl in combination with Myc-PI3KC2β WT or DN, or empty vector. Immunoprecipitates prepared with anti-Dbl antibodies were analysed for GEF activity towards recombinant RhoA.

### Rho GTPases Activation Assay

NIH3T3-V, -C2β-WT, -C2β-DN cells were grown in 10% FCS to confluence or they were made quiescent by culturing them in serum-free medium (0.1%) for 24 h. After starvation they were stimulated either with 1nm PDGF or 1 µM LPA for 2 min., as indicated in the figure legends. Afterwards, the cells were washed with ice-cold PBS supplemented with 1 mM MgCl_2_ and immediately lysed in ice-cold Triton 1% lysis buffer (50 mM Tris-HCl pH 7.5, 1% Triton X-100, 0.5% sodium deoxycholate, 0.1% SDS, 500 mM NaCl, 10 mM MgCl_2_, 10 µM leupeptin, 10 µM pepstatin and 1 mM PMSF). The cells were rapidly scraped off the plates and the crude lysates were transferred to pre-chilled Eppendorf tubes and centrifuged at 16,060×g for 5 min. at 4°C. Protein concentration was determined (Bradford assay, Pierce) and equal amounts of lysates were immediately supplemented with GST-PAK-CRIB or GST-rhotekin for detection of activated Rac1 and RhoA, respectively. GST-fusion protein (20–30 µg) on glutathione beads was added to the supernatants and the tubes were incubated with constant rotation at 4°C for 30 min. The beads were thereafter washed 4x with ice-cold washing buffer (50 mM Tris-HCl pH 7.5, 1% Triton X-100, 150 mM NaCl, 10 mM MgCl_2_, µM leupeptin, 10 µM pepstatin and 1 mM PMSF). SDS-PAGE sample buffer was added. Samples were heated at 95°C for 3 min. and subjected to SDS-PAGE and western blot analysis.

**Figure 6:**
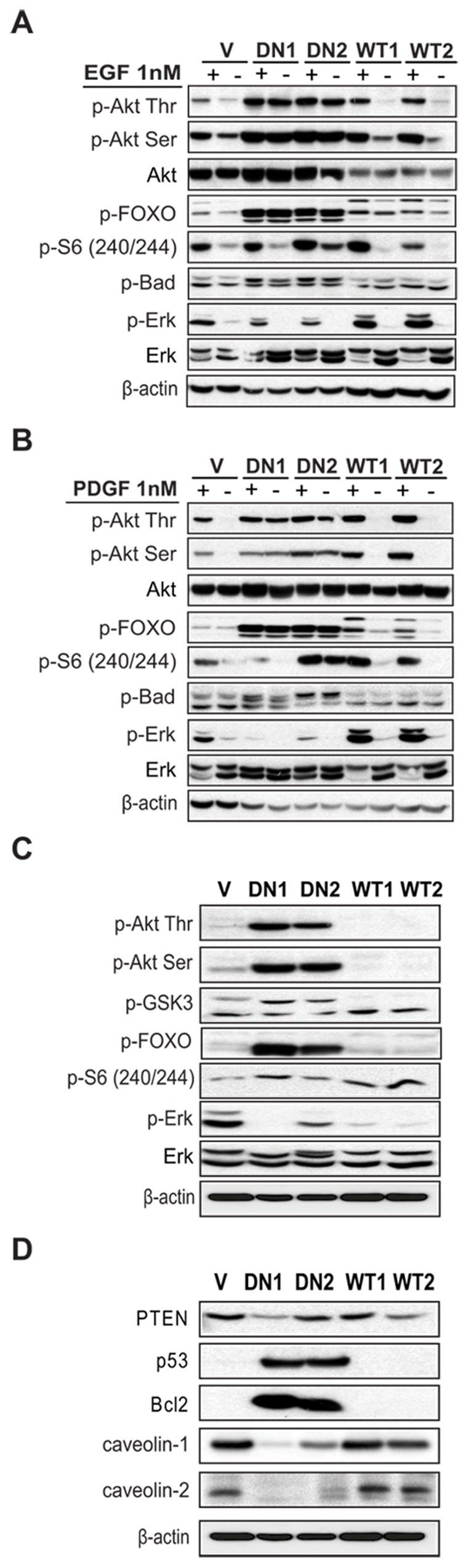
PI3KC2β protects NIH3T3 cells against anoikis. (A) NIH3T3-V, -C2β-DN and -C2β-WT cells were plated in presence of serum on ultra-low attachment matrix. After 16 h Caspase 3/7 activity was measured as readout for detachment-induced apoptosis (anoikis). Data are mean ± SD of two independent experiments. WT1, −2 and DN1, −2 indicate individual clones. (B) NIH3T3 cells transiently transfected with the wild-type Myc-PI3KC2β, constitutively active form of GFP-fused RhoA protein (CA-RhoA-GFP) or myristylated (Akt-myr) were plated 48 h post-transfection on ultra-low attachment matrix. 16 h later Caspase 3/7 activation was analysed as readout for anoikis. Data are mean ± SD of three independent experiments. Expression level of the transfected proteins and associated signalling was analysed 48 h post-transfection by immunoblotting with indicated antibodies. (C) NIH3T3-C2β-WT cells were transiently transfected with dominant-negative RhoA-GFP (DN-Rho-GFP) or control vector (V). 48 h post-transfection cells were plated on ultra-low attachment matrix and caspase 3/7 activation was measured. Data are mean ± SD of three independent experiments. 48 h post-transfection expression level of the transfected proteins was analysed by western blot with indicated antibodies.

### GEF Activity Aassays

HEK293 cells were transiently transfected with plasmids as described above. Cell lystaes were prepared as above and immunoprecipitated with anti-Dbl antibodies and Protein A-Sepharose beads. The immunoprecipitates were washed twice with lysis buffer and once with GEF reaction buffer (20 mM Tris-HCl pH 7.6, 0.5% Triton X-100, 100 mM NaCl, 10 mM MgCl_2_, 1 mM DTT) and resuspended in GEF reaction buffer on ice. The reaction was started by the addition of 1 mM GTPγS and 50 µg/ml purified recombinant RhoA and incubated for 30 min at room temperature. The samples were then placed on ice and supernatants collected. Active RhoA was isolated from the supernatants by using GST-rhotekin, as described above and samples were analysed by SDS-PAGE and western blot.

**Figure 7:**
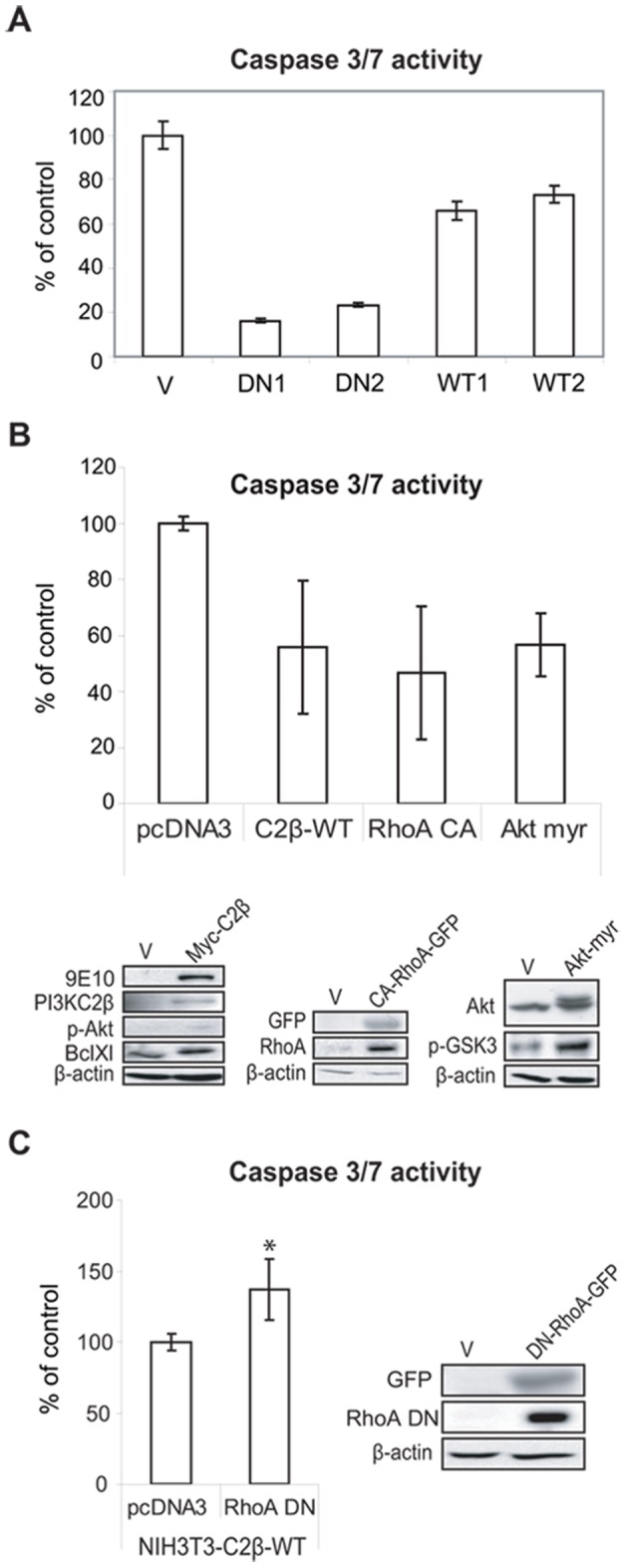
PI3KC2β increases MAPK and Akt signalling downstream of EGFR and PDGFR. (A) and (B) NIH3T3-V, -C2β-DN and -C2β-WT cells were serum-deprived overnight and stimulated for 10 min. with EGF (A) or PDGF (B) as indicated. Cell lysates were analysed by immunobloting and MAPK and Akt pathway activation was assessed with indicated phospho-specific antibodies. (C) and (D) Lysates of NIH3T3-V, -C2β-DN and -C2β-WT cells grown in 10% FCS were analysed for activated signalling molecules implicated in the cell cycle control and caveolae formation such as p53, Bcl2, PTEN and caveolin 1, −2. WT1, −2 and DN1, −2 indicate individual clones.

### Pull-down Assays for PI3KC2β/Dbl/Grb2 Interaction

After 48 h of culture in 10% FCS medium, untransfected and transfected with HA-proto-Dbl or pcDNA3 empty vector HEK293 cells were washed with ice-cold PBS and lysed on ice for 20 min. in Triton 1% (50 mM Tris-HCl pH 7.5, 150 mM NaCl, 1 mM EDTA, 0.5 mM EGTA, 1% Triton X-100, 0.5% NP-40, 1 mM DTT added before use) lysis buffer supplemented with 7×concentrated stock of protease inhibitors (Complete Mini, Roche) and phosphatase inhibitors (1 mM NaF, 1 mM Na_3_VO_4_ and 10 mM β-glycerophosphate). The crude lysates were centrifuged at 16,000×*g* for 25 min. at 4°C. Protein concentration was determined (BCA, Pierce) and equal amounts of lysates were immediately supplemented with GST-NT-C2β, GST-(CT)C2-C2β or GST-Grb2 immobilized on gluthatione beads. The tubes were incubated with constant rotation for 2 h at 4°C. The beads were thereafter washed four times with ice-cold lysis buffer. SDS-PAGE sample buffer was added. The samples were heated at 95°C for 3 min. and subjected to SDS-PAGE and western blot analysis for detection of interaction with Dbl.

In the case of Dbl domains interaction with PI3KC2β, COS-1 cells were co-transfected with wild-type PI3KC2β and GST-tagged Dbl domains (NT-, spectrin-, onco-, PH) or pcDNA3 epmty vector and HA-proto-Dbl as control. 48 h post-transfection cells were washed with ice-cold PBS and lysed on ice for 20 min. in Triton 1% lysis buffer supplemented with protease and phosphatase inhibitors (as described above). The crude lysates were centrifuged at 16,000×*g* for 25 min. at 4°C. Protein concentration was determined (BCA, Pierce) and equal amounts of lysates were supplemented with gluthatione-sepharose beads (GE Healthcare). The tubes were incubated with constant rotation for 2 h at 4°C. The beads were thereafter washed four times with ice-cold lysis buffer. SDS-PAGE sample buffer was added. The samples were heated at 95°C for 3 min. and subjected to SDS-PAGE and western blot analysis.

### In Vitro Binding Assay

To test the PI3KC2β association to Dbl spectrin- and PH-domains an *in vitro* binding assay, which utilized GST-tagged Dbl spectrin- and PH- domains and GST alone immobilized on gluthatione-speharose beads was performed. Immobilized proteins were incubated *in vitro* with soluble Grb2- and NT-C2β- domain in 1% Brij 96 (50 mM Tris.Cl pH 7.4, 150 mM NaCl, 1 mM EDTA, 0.5 mM EGTA, 1% Brij96) lysis buffer supplemented with 7×concentrated stock of protease (Complete Mini, Roche) and phosphatase inhibitors (1 mM sodium fluoride, 1 mM sodium orthovanadate, 10 mM β-glycerophosphate). Soluble Grb2- and NT-C2β-domain were obtained from GST-fused Grb2 and GST-N-terminal PI3KC2β domain as a result of digestion with thrombin. Following constant rotation for 2 h, at 4°C, the complexes were washed 3x with Brij 96 1% lysis buffer. SDS-PAGE sample buffer was added and the samples were heated at 95°C for 5 min. and subjected to SDS-PAGE followed by western blot analysis.

### Cell Proliferation

Cell counting experiments based on Trypan Blue exclusion were performed. NIH3T3-V, -C2β-WT, -C2β-DN cells were grown in 12-well plates at a density 20 cells/µl in the presence of either 10% or 0.5% FCS. After 48 h cells were washed once with phosphate-buffered saline (PBS) and trypsinized. When needed clumps within the cell suspension were disaggregated by passing the well content 5 times through a 20-gauge needle and the number of viable cells was determined by Trypan Blue exclusion, using a haemocytometer. Experiment was performed in triplicate.

### Wound Healing Assay

For wound healing assays, NIH3T3-V, -C2β-WT, -C2β-DN cells were plated in 12-well culture plates in complete medium and grown to confluency. Where indicated, confluent cells were serum-deprived overnight and treated with 10 nM PDGF. A wound was done by making a scratch on the cells monolayer with a 200 µl tip. The migration rate was monitored for 16 h by phase contrast microscopy (Leica DM IRBE Inverse, Widefield) equipped with a temperature controller. The wounded cell monolayers were photographed and quantitative analysis was prepared for each experiment. The wounded area was defined in each image by positioning lines in correspondence of the original scratch and wound closure distance was analysed by micrometer measurements.

### Cell Death (Anoikis) Assay

NIH3T3-V, NIH3T3-C2β-WT, NIH3T3-C2β-DN cells or NIH3T3 and NIH3T3-C2β-WT cells transiently transfected (Lipofectamine, Invitrogen) with pcDNA3 empty vector, PI3KC2β-WT, constitutively active or dominant-negative form of GFP-fused RhoA and myristylated Akt were used. Cells were grown for 48 h in attachment then trypsinized and washed once with DMEM/10% FCS. The cells were then placed in Ultralow attachment 96-well plates (Corning) at 2×10^5^ cells/well in 100 µl DMEM containing 10% FCS at 37°/5% CO_2._ After 16 h detachment-induced apoptosis was analysed based on the caspase 3/7 activation using the Caspase-Glo 3/7 Assay (Promega), according to manufacturer’s instruction.

### Immunofluorescence

Cells were seeded on glass coverslips and grown for 48 h. Coverslips were rinsed in PBS and fixed with 4% paraformaldehyde. Prior to permeabilisation with 0.2% Triton X-100, cells were incubated with 0.1M glycine. After blocking in 2% horse-serum immunostaining was performed with an anti-PI3KC2β antibody followed by FITC-conjugated goat anti-rabbit antibody and Alexa Fluor 555 conjugated phalloidin (Invitrogen). Coverslips were mounted in Mowiol (Centre for Microscopy and Image Analysis, Zurich) and cells were examined by confocal laser scanning microscopy (Leica SP1, Centre for Microscopy and Image Analysis, Zurich).

### Luciferase Reporter Assays

Transient transfections of NIH3T3 cells were carried out using Lipofectamine (Invitrogen) according to the manufacturer’s protocol. Around 5×10^4^ cells per well in 12-well plate format were transfected with 25 ng of p3DA-Luc and 50 ng of pRL-TK (Promega, Madison, WI). After transfection cells were maintained in 0.5% FCS for 24 h before stimulation with 15% serum for seven hours. Firefly luciferase activity was normalized to pRL-TK luciferase activity. Figures show mean ± SD of three independent experiments.

### Statistical Analysis

Analysis of variance and Bonferroni multiple comparison test or Kruskal-Wallis non-parametric analysis of variance test were used to assess statistical significance of differences between groups. P values <0.05 were considered as significant.

## Results

### PI3KC2β Interacts with Dbl

The small GTPases Rac and RhoA are regulated by proteins modulating their GTPase activity. We and others reported the interaction of PI3KC2β with RhoGEFs such as Eps8 and intersectin [17], [40]. To elucidate the molecular mechanism of PI3KC2β–dependent Rac and RhoA activation in NIH3T3 cells, we investigated the binding of PI3KC2β to a panel of RhoGEFs family members. We performed co-immunoprecipitation studies using an anti-PI3KC2β and an anti-epitope-Glu-tag (EE) antibody with lysates of NIH3T3 cells stably expressing Glu-tagged wild-type (WT) PI3KC2β form. As shown in Fig. 1A, we detected a binding of the recombinant PI3KC2β with endogenously expressed RhoGEF Dbl, which belong to one of the best studied regulators of RhoGTPases [36]. Our further analysis showed that PI3KC2β co-immunoprecipitated Grb2 and Src (Fig. 1A). Grb2 has been reported to serve as an adaptor protein for PI3KC2β downstream of the EGFR in HEK293 cells [12], whereas Src is involved in EGFR and PDGFR downstream signalling in NIH3T3 fibroblasts [41]. The results revealed an endogenous PI3KC2β/Dbl complex in the NIH3T3 parental cell line (Fig. 1B). To confirm our findings, we selected IMR5, a neuroblastoma cancer cell line, which expresses relatively high levels of PI3KC2β and Dbl, and performed co-immunoprecipitation with anti-Dbl and anti-PI3KC2β antibodies. As depicted in Figure 1B, PI3KC2β associated with Dbl in the IMR5 cells confirming the formation of an endogenous protein complex. We were further interested in studying the molecular mechanism of PI3KC2β/Dbl complex assembly. Pull down experiments with GST-fused PI3KC2β N-terminal and C2 C-terminal domain and lysates of HEK293 cells transfected with HA-tagged proto-Dbl revealed that the N-terminal sequence of the PI3K is involved in the interaction with Dbl (Fig. 1C). The N terminus of PI3KC2β (in particular proline-rich regions) was previously shown to mediate PI3KC2β association with the activated EGF receptor through the Grb2 adaptor protein [12]. Interestingly, our pull down experiments with purified GST-fused Grb2 and lysates of HEK293 cells transfected with HA-tagged proto-Dbl showed binding of Grb2 to Dbl, and confirmed the interaction with PI3KC2β (Fig. 1D). Additionaly, Grb2 complex formation with endogenous Dbl was detected in untransfected HEK293 cells, which confirms the relevance of this interaction. These findings demonstrate the formation of a multi-protein complex comprising PI3KC2β, Grb2 and Dbl, which may be functional in normal cells (NIH3T3), as well as in cancer cells (IMR5). Whether the protein-protein interactions in this complex are direct or mediated by other possible interaction partners was further elucidated in an *in vitro* binding assay.

### Dbl RhoGEF Domains Bind to PI3KC2β with Different Affinities

Considering the multi-domain structure of Dbl, we were interested in uncovering which part of the protein sequence is involved in the interaction with PI3KC2β. Pull down experiments with glutathione-sepharose beads from COS-1 cells co-transfected with GST-fused Dbl N-terminal-, spectrin-, onco-, PH- mutants and wild-type PI3KC2β showed a prominent interaction of the kinase with the Dbl spectrin- and PH- domain (Fig. 2). On the other hand, none of the Dbl domains bound to Grb2, whereas Grb2 associated with full-length HA-proto-Dbl immunoprecipitated with anti-Dbl antibody and immobilized on beads. The N-terminal spectrin- and C-terminal PH- domain of Dbl were previously shown to associate with the chaperone Hsc70. This interaction results in Dbl adopting an inactive conformation, which in turn blocks an access of GTPases to its DH- catalytic domain [31], [33]. Furthermore, it was suggested that the spectrin domain regulates the level, localization, and activity of proto-Dbl through the interaction with Hsc70-Hsp90 and the ubiquitin ligase CHIP [32]. However, further investigations are required to exactly assess the contribution of PI3KC2β to the regulation of Dbl activity.

The binding of Dbl spectrin and PH domains to PI3KC2β in co-transfected COS-1 cells led us to test whether this interaction is direct. Therefore, an *in vitro* binding assay was performed, which used recombinant GST-tagged spectrin- and PH- Dbl domains immobilized on gluthatione-sepharose beads together with the soluble N-terminal region of PI3KC2βor/and Grb2– expressed and purified in *E.coli* (Fig. S1). In the case of NT-PI3KC2β, we did not observe an interaction with Dbl. On the other hand, Grb2 showed an interaction, but due to detectable binding with GST control, we considered it as un-specific. The lack of association of the NT-PI3KC2β and Grb2 with Dbl domains suggests that other factors are required for the interaction in living cells (Fig. S1).

### Overexpression of PI3KC2β is Sufficient to Induce Cell Morphology Changes in a Kinase-dependent Manner

We and others have reported that PI3KC2β interacts with different receptor tyrosine kinases such as EGFR, PDGFR and c-Kit [9], [10], [12]. We aimed to investigate the biological importance of PI3KC2β in NIH3T3 mouse fibroblast cells. Since these cells have very low levels of endogenous PI3KC2β expression, we established NIH3T3 clones stably expressing an epitope wild-type (WT) and a mutant kinase-dead (DN) PI3KC2β form. NIH3T3 cells stably transfected with an empty pBabeNeo vector were used as control. We utilised a mutant that we previously reported to eliminate PI3KC2β-associated lipid kinase activity by mutation of the highly conserved aspartate (DFG) to an alanine residue in the activation domain (D1213A, PI3KC2β-DN) [17].

Western blot analysis confirmed the expression of each of the constructs (Fig. 3A). For further experiments clones with high recombinant protein expression were selected. Interestingly, we observed striking morphological differences between the cell clones differentially expressing either the WT or DN PI3KC2β. In comparison to control cells, NIH3T3-C2β-WT cells displayed increased cell spreading with strong stress fibres assembly and increased membrane ruffling revealed by F-actin staining (Fig. 3B), whereas the clones expressing the kinase-dead form of PI3KC2β did not display F-actin polymerisation and presented rounded phenotype with a strongly reduced cell spreading (Fig. 3B). Staining for PI3KC2β showed cytosolic distribution with additional localisation of the protein to cell extensions and cell-cell contacts (Fig. 3B). Stimulation with PDGF led to more pronounced membrane localisation of PI3KC2β protein in NIH3T3-C2β-WT cells (data not shown), revealing recruitment to the activated PDGF receptor in the cell membrane as reported [9]. In line with our previous report [17], these data demonstrate that the lipid kinase activity of PI3KC2β plays a role in modulating the actin cytoskeleton in NIH3T3 cells.

### PI3KC2β Regulates Rac and RhoA Activity

Due to the observation, that NIH3T3-C2β-WT cells show increased spreading and membrane ruffling, two phenotypes related to the small GTPases RhoA and Rac1 [24], we analysed RhoA activity in serum-supplemented conditions and found highly elevated basal levels of GTP-loaded RhoA in NIH3T3-C2β-WT cells in comparison to control cells (Fig. 4A). Based on recent observation that PI3KC2β modulates cell migration in response to lysophosphatidic acid (LPA) [16], we treated serum-starved cells with LPA, a commonly used RhoA activator [42]. NIH3T3-C2β-WT cells showed increased RhoA activation, but also higher basal activity in low serum, compared to control cells (Fig. 4A). NIH3T3-C2β-DN cells displayed only a minimal response to LPA treatment (Fig. 4A). To confirm these observations we used a different approach, making use of the fact that RhoA activates a MADS-Box transcription factor termed serum response factor (SRF) [43]. SRF controls growth factor regulated immediate-early genes such as c-*fos* and cytoskeletal actin [44]. RhoA downstream targets such as mDia and LIM kinase 1 effectively regulate SRF by modulating the activity of a SRF co-factor termed MAL [45]. MAL is constitutively bound to monomeric actin and thus sequestered in the cytosol. Activation of RhoA in response to serum leads to actin polymerisation, MAL release from G-actin and its translocation to the nucleus, where it binds and activates SRF [45]. In a transactivation luciferase assay we determined up to 5-fold MAL-dependent SRF response in NIH3T3-C2β-WT cells after serum stimulation as compared to control cells (Fig. 4B). In contrast, NIH3T3-C2β-DN cells showed only minimal SRF activation (Fig. 4B).

We next analysed Rac activity in serum-supplemented conditions. NIH3T3-C2β-WT cells showed significantly elevated Rac GTP loading, whereas the three DN clones tested had reduced Rac activity compared to control cells (Fig. 4C). Next we investigated Rac activation downstream of the PDGFR. We found strongly increased Rac GTP levels upon PDGF stimulation in NIH3T3-C2β-WT cells compared to control cells (Fig. 4D). Phosphorylation of the downstream Rac target SAPK/JNK was strongly elevated as well (Fig. 4D). Interestingly, in NIH3T3-C2β-DN we observed down to 50% reduced Rac activation and accordingly very weak SAPK/JNK phosphorylation (Fig. 4D). Taken together, these results indicate that PI3KC2β is implicated in the LPA and serum dependent activation of RhoA and in the Rac activation upon PDGF treatment.

### The Assembly of the Dbl - PI3KC2β Complex is not Modulated by PI3K Activity or Cell Stimulation with EGF or PDGF

The data presented above suggested that the catalytic activity of PI3KC2β may modulate activation of Rho/Rac proteins via Dbl. In order to further investigate the importance of PI3KC2β kinase activity for its interaction with Dbl, we assessed the ability of PI3KC2β WT and DN to interact with Dbl in transiently transfected HEK293 cells and stably transfected NIH3T3 cells. In HEK293 cells, both PI3KC2β WT and DN interacted with endogenous or ectopically expressed (HA-tag) Dbl in a similar manner (Fig. 5A). This observation was confirmed in NIH3T3-C2β-WT and NIH3T3-C2β-DN cells (Fig. 5B).

We next assessed whether the Dbl - PI3KC2β complex was constitutively assembled, or induced by cell stimulation with polypeptide growth factors. The Dbl - PI3KC2β complex could be detected in serum-starved NIH3T3-C2β-WT cells, and the interaction was apparently not increased by cell stimulation with EGF or PDGF from 5′ up to 30′ (Fig. 5C).

Finally, we investigated whether the catalytic activity of PI3KC2β contributes to the activation of Dbl GEF activity. In transiently transfected HEK293 cells, ectopic expression of PI3KC2β WT or DN did not alter the GEF activity of endogenous Dbl towards RhoA (Fig. 5D). Taken together, these results indicate that PI3KC2β lipid kinase activity is not required for its association with Dbl, and does not modulate Dbl activity measured *in vitro*.

### PI3KC2β Protects NIH3T3 Cells from Detachment-induced Death (Anoikis)

Forkhead fox O (FOXO) family of transcription factors have been reported to control the expression of caveolin-1 [46]. Phosphorylated FOXO remains in the cytosol [47] and is not able to activate Caveolin 1 transcription. Based on these reports we investigated Caveolin 1 and 2 expression and found strong protein downregulation of both proteins (Fig. 6D). These two isoforms are crucial for the proper formation and function of caveolae: membrane invaginations serving as signalling platforms on the cell surface and are often disassembled in cancer cells [48]. Caveolin 1 deleted cells become resistant to detachment-induced death [49] and caveolin 1 deletion is sufficient to drive cell transformation in NIH3T3 cells [50]. Interestingly, in NIH3T3-C2β-DN cells we observed strongly reduced matrix attachment to collagen and fibronectin and loss of contact inhibition, thus NIH3T3-C2β-DN cells were able to proliferate in multi-layers after reaching confluence without undergoing apoptosis (data not shown). Surprisingly, we found that p53 was strongly elevated in these cells (Fig. 6D). However the anti-apoptotic Bcl-2 protein showed also strongly increased expression (Fig. 6D). These protein deregulations revealed a pattern reported for cells protected from anoikis [51]. We analysed cell death induction by plating the cells on ultra-low attachment matrix. After 16 h incubation NIH3T3-C2β-DN cells showed very robust apoptosis protection measured by Caspase 3/7 activation, nevertheless the NIH3T3-C2β-WT cells had also lower apoptosis induction compared to control cells (Fig. 7A). Since inhibition of RhoA results in loss of substrate adhesion and severe cell retraction [24], we hypothesised that due to the long lasting clonal selection, cells with high kinase-dead PI3KC2β expression and hence attenuated RhoA function will gain survival advantage only under conditions when are able to upregulate signalling pathways protecting them from the RhoA-dependent lost of cell anchorage and the following apoptosis induction.

We reported that PI3KC2β protects A-431 epithelial carcinoma cells from anoikis, however the mechanism remain elusive [17]. To test the hypothesis that wild-type PI3KC2β protects cells from anoikis in a RhoA dependent manner, we performed transiently expression of wild-type PI3KC2β, constitutively active RhoA and myristoylated Akt as a positive control in NIH3T3 cells. All three proteins were able to decrease significantly Caspase 3/7 activation in cells plated on ultra-low attachment matrix for 16 h, compared to NIH3T3 cells transfected with an empty vector (Fig. 7B). Moreover, transient transfection of NIH3T3-C2β-WT cells with a dominant-negative RhoA construct led to an increase in apoptosis induction compared to parental cells (Fig. 7C). These findings reveal an important role of PI3KC2β in protecting cells from detachment-induced apoptosis in a RhoA-dependent manner.

### PI3KC2β Expression Increases the Activation of EGFR and PDGFR Downstream Signalling

The recruitment and activation of PI3KC2β downstream of the EGFR and the PDGFR has been reported [9], [12]. In agreement with these reports, the results discussed above pointed to a PDGFR related PI3KC2β function in cell migratory responses. Therefore, we aimed to investigate Akt and Erk signalling pathway activation downstream of these two receptors. In line to our previous findings [10], elevated expression of wild-type PI3KC2β increased Akt pathway activation upon EGF and PDGF treatment, illustrated by increase in serine and threonine Akt phosphorylation, FOXO and S6 protein phosphorylation (Fig. 6A and B). Moreover, the extracellular regulated kinase (Erk1) showed also increased phosphorylation, compared to control cells. Surprisingly, we found very high basal Akt and FOXO phosphorylation levels in NIH3T3-C2β-DN cells. Moreover, EGF or PDGF stimulation did not further increase the Akt and FOXO phosphorylation (Fig. 6A and B). On the contrary, Erk activation was strongly abolished in these cells and the response to PDGF was clearly weaker than to EGF (Fig. 6A and B).

In order to understand how these signalling differences contribute to the observed cytoskeletal rearrangements, we further studied signalling in cells grown in serum-supplemented conditions. Very high basal Akt and FOXO phosphorylation was detected in the NIH3T3-C2β-DN cells, however we did not find any striking differences in Akt and FOXO phosphorylation between NIH3T3-C2β-WT and control cells (Fig. 6C). Also the expression of the negative regulator of PI3K signalling PTEN was not consistently decreased in the NIH3T3-C2β-DN cells under study (Fig. 6D). However, we observed an induction of p53 expression in the NIH3T3-C2β-DN cells (Fig. 6D). In addition these cells displayed increased Bcl-2 expression and reduced expression of caveolin-1 and caveolin-2 (Fig. 6D). Thus it appears that NIH3T3-C2β-DN cells display prominent changes in the expression of major regulators of apoptosis and tumour suppressors. It is conceivable that enforced expression of dominant negative PI3KC2β in NIH3T3 cells leads to p53 induction and anoikis and that the surviving NIH3T3-C2β-DN cells have managed to escape apoptosis during the selection process by up-regulating Bcl-2 expression and activating the Akt pathway. The down-regulation of caveolin expression in NIH3T3-C2β-DN cells may contribute to the constitutive activation of the Akt pathway, by relieving an inhibitory effect on RTK signalling. Collectively, our data reveal the implication of PI3KC2β in the activation of the EGFR- and PDGFR-dependent Akt and Erk signalling cascades.

### PI3KC2β Mediates Migratory Response Downstream of the PDGFR

Next, we attempted to analyse the migratory properties in NIH3T3 cells with deregulated PI3KC2β expression. We performed a wound healing assay under serum-supplemented conditions and in cells upon PDGF stimulation. Compared to control cells neither PI3KC2β-WT nor PI3KC2β-DN had a significant impact on the migratory cell speed in 10% serum (Fig. S2A). However, when cells were starved for 24 hours and then PDGF was added, we observed very strong migratory delay down to 80% in NIH3T3-C2β-DN cells (Fig. S2A). Interestingly, NIH3T3-C2β-WT cells were also not able to fully respond and migrated at 60% speed of control cells. These effects were not due to a different proliferation status, since NIH3T3-C2β-WT cells showed even slightly increased proliferation in serum. Surprisingly, NIH3T3-C2β-DN cells showed nearly doubled cell proliferation in low serum (Fig. S2B). These experiments reveal a PI3KC2β-dependent migratory response downstream of the activated PDGF receptor in NIH3T3 cells.

## Discussion

Our analysis of the interacting molecules of PI3KC2β in NIH3T3 fibroblasts revealed a novel signalling complex implicated in RhoA and Rac1 activation. We identified the RhoGEF Dbl as a novel interaction partner of PI3KC2β and we hypothesise that, in collaboration with Dbl, PI3KC2β can modulate RhoA and Rac1 activity and thus control cytoskeletal rearrangements, cell motility and protection against anoikis. Dbl is a crucial RhoGEF with activity towards Cdc42, RhoA and Rac1 [52]. It was also the first mammalian RhoGEF to be identified [26], but the mechanism of its activation is still not completely understood. In order to relieve its intrinsic auto-inhibitory activity, a physical interaction between the GEF’s N-terminal spectrin- and C-terminal PH-domain has to be released. This change in the inactive conformation of the protein can be triggered by diverse regulatory mechanisms including phosphorylation, or interactions with some other proteins. In our studies, the prominent binding of PI3KC2β to the spectrin- and PH- domain of Dbl was similar to the results reported for Dbl binding to the chaperone Hsc70 [31]. Hsc70 together with Hsp90 and the ubiquitin ligase CHIP keep Dbl activity in check by stabilising its inactive conformation. An *in vitro* examination of the molecular mechanism of PI3KC2β/Dbl complex formation, however, revealed that neither the N-terminal sequence of the PI3K, nor Grb2, directly bound to Dbl, although both molecules were present in the PI3KC2β complex isolated from living cells. Formation of the Dbl/PI3KC2β complex was observed in serum-starved cells and the association was not further promoted by EGF or PDGF stimulation. In addition, we did not find evidence for a role of PI3KC2β in the activation of Dbl GEF activity, at least when measuring its activity *in vitro*. Therefore, other molecules need to be present in the cells, which mediate the interaction of the class II PI3K with Dbl and Dbl activation, resulting in the observed phenotypes in the NIH3T3 cells. The identity of these putative additional binding partners involved in the Dbl/PI3KC2β complex is briefly discussed below.

Grb2 has been reported to be crucial for the EGF-dependent Dbl phosphorylation and subsequent GEF activation towards RhoA and Cdc42 [53], [54]. These findings support our observation that Grb2 is present in the Dbl/PI3KC2β complex in NIH3T3 cells. Dbl phosphorylation is catalysed by the non-receptor tyrosine kinase Ack1 [54], a widely expressed protein which is recruited and activated downstream of the EGFR and PDGFR via Grb2 [55]. Furthermore, Dbl binds to newly generated phosphoinositides (mainly PI(3,4,5)P_3_ and PI(4,5)P_2_), via its PH domain thereby localizing to the plasma membrane [56]. Considering the potential of Class II PI3K to generate the same spectrum of phosphoinositides as Class I PI3K [7], [9], [14], this model provides a possibility for PI3KC2β to build up a complex consisting of Grb2, ACK1 and Dbl, which may be important for RhoA and Rac activation in serum-supplemented conditions and Rac activation upon EGF or PDGF stimulation. After EGF or PDGF stimulation, the Dbl/PI3KC2β complex may be recruited to the plasma membrane via Grb2 and generates PI(3,4,5)P_3_, which binds to the PH domain of Dbl. Grb2, on the other hand, may bind to ACK11 complexed with Cdc42. Being in close proximity, ACK1 could therefore bind to Dbl. Ack1 could phosphorylate Dbl only when in complex with Grb2 and Cdc42, which then become activated and in turn activates RhoA and Rac leading to stress fibres and membrane ruffles formation. Further investigations of the phosphorylation status of Dbl in the NIH3T3 cells over-expressing PI3KC2β are needed to confirm this model.

Surprisingly, the increased basal and PDGF-induced Rac1 activity in NIH3T3 cells expressing wild-type PI3KC2β did not correspond to increased wound healing properties, a response which can be explained by the fact that active RhoA and the generation of focal adhesions and stress fibres significantly slow down cell migration [57]. NIH3T3 cells transfected with dominant negative PI3KC2β cells failed to activate Rac1 after PDGF stimulation. This became more evident when looking at the completely abolished wound healing properties of the cells after PDGF treatment, once again confirming the importance of PI3KC2β-dependent PDGFR signalling. The RhoA-activating function of PI3KC2β also had inhibitory effects on detachment-induced apoptosis. We observed this outcome in both NIH3T3 cells stably expressing wild type PI3KC2β and in NIH3T3 cells transiently transfected with the wild type PI3KC2β. Moreover, in both systems, caspase activation was reduced down to ∼65% of its original activity after plating the cells under ultra-low attachment conditions. The same effect was observed after transient overexpression of constitutively active RhoA in NIH3T3 cells. These results are in agreement with a recent report showing that active RhoA was able to rescue NIH3T3 cells from detachment-induced apoptosis [58]. This anti-apoptotic effect under stress conditions could play also a role under normal conditions as we observed slightly, but significant, increase in cell proliferation in NIH3T3 cells expressing wild type PI3KC2β, compared to control cells. Even ROCK, a downstream target of RhoA has been widely accepted as mediator of apoptosis by inducing characteristic morphological changes, including cell contraction and dynamic membrane blebbing, RhoA seems to be excluded from this signalling cascade [59]. Here we provide further evidence that sustained RhoA activation provides NIH3T3 cells with pro-survival signals and enables anchorage-independent cell survival, one of the first phenomenon described for oncogenic Rho proteins [60].

Together, our data show for the first time that overexpression of PI3KC2β in NIH3T3 cells is sufficient to induce RhoA and Rac activation and thus to promote cell transforming properties, reported for both RhoA and Rac [61], [62]. We have further identified the Dbl RhoGEF as a new interaction partner for PI3KC2β. Previous reports [10], [17] and unpublished observations have revealed that PI3KC2β is overexpressed in different human cancer cell lines and primary tumour samples, and modulates cell survival and chemotherapy resistance [63]. Together with the present report we propose a model in which PI3KC2β can contribute to the protection of cells from anoikis and increase Rho/Rac, Akt and Erk signalling downstream of the EGFR and PDGFR. This signalling pathway may therefore contribute to the malignant progression of human cancers.

## Supporting Information

Figure S1 PI3KC2β and Grb2 do not associate directly with Dbl. (A) GST-tagged and gluthatione-sepharose beads conjugated spectrin- and PH- Dbl domains, and GST as control, were incubated *in vitro* with GST-truncated soluble PI3KC2β N-terminal domain (NT-C2β) (∼35 kDa) and Grb2 (26 kDa). To detect an interaction wih Dbl domains samples were subjected to immunoblotting with indicated antibodies. Experiment was repeated four times with similar results, independently of buffer used for the reaction (Brij96 or Triton 1%). One representative blot is shown (Brij96 1% used for the incubation). (B) Control *in vitro* reactions, which utilized immobilized on gluthatione-sepharose beads GST-tagged NT-C2β domain and Grb2, and GST as control, were incubated *in vitro* with GST-truncated soluble NT-C2β domain (∼35 kDa) and Grb2 (26 kDa). Samples were subjected to SDS-PAGE analysis and the interaction between NT-C2β and Grb2 was detected with anti-GST antibody by western blot analysis. (C) Representative immunoblot of Dbl domains separated on SDS-PAGE and analysed by wester blot with anti-GST antibody.
(TIF)

Figure S2 PI3KC2β regulates wound healing properties. (A) Wound healing assay in NIH3T3-V, -C2β-DN and -C2β-WT cells, which were grown in 10% FCS or were serum-starved over night and stimulated with 10 nM PDGF. Migration rate was monitored for 16 h by phase contrast microscopy. Graph presents quantitative analysis of time-lapse microscopy pictures. Data are mean ± SD of two independent experiments. (B) Cell proliferation of NIH3T3-V, -C2β-DN and -C2β-WT cells, which were growing for 48 h either in 10% or 0.5% FCS was performed. The number of viable cells was determined based on Trypan Blue exclusion and cell counting. Data are mean ± SD of three independent experiments. *, p<0.05.
(TIF)

## References

[1] Katso R, Okkenhaug K, Ahmadi K, White S, Timms J, et al (2001) Cellular function of phosphoinositide 3-kinases: implications for development, homeostasis, and cancer. Annu Rev Cell Dev Biol 17: 615–675. 11687500 10.1146/annurev.cellbio.17.1.615

[2] Wymann MP, Bjorklof K, Calvez R, Finan P, Thomast M, et al (2003) Phosphoinositide 3-kinase gamma: a key modulator in inflammation and allergy. Biochem Soc Trans 31: 275–280. 12546701 10.1042/bst0310275

[3] Sasaki T, Suzuki A, Sasaki J, Penninger JM (2002) Phosphoinositide 3-kinases in immunity: lessons from knockout mice. J Biochem 131: 495–501. 11926985 10.1093/oxfordjournals.jbchem.a003126

[4] DiNitto JP, Cronin TC, Lambright DG (2003) Membrane recognition and targeting by lipid-binding domains. Sci STKE 2003: re16. 14679290 10.1126/stke.2132003re16

[5] Engelman JA, Luo J, Cantley LC (2006) The evolution of phosphatidylinositol 3-kinases as regulators of growth and metabolism. Nat Rev Genet 7: 606–619. 16847462 10.1038/nrg1879

[6] Schmidt A, Hall A (2002) Guanine nucleotide exchange factors for Rho GTPases: turning on the switch. Genes Dev 16: 1587–1609. 12101119 10.1101/gad.1003302

[7] Arcaro A, Volinia S, Zvelebil MJ, Stein R, Watton SJ, et al (1998) Human phosphoinositide 3-kinase C2beta, the role of calcium and the C2 domain in enzyme activity. J Biol Chem 273: 33082–33090. 9830063 10.1074/jbc.273.49.33082

[8] Domin J, Pages F, Volinia S, Rittenhouse SE, Zvelebil MJ, et al (1997) Cloning of a human phosphoinositide 3-kinase with a C2 domain that displays reduced sensitivity to the inhibitor wortmannin. Biochem J 326 (Pt 1): 139–147. 10.1042/bj3260139 PMC1218647 9337861

[9] Arcaro A, Zvelebil MJ, Wallasch C, Ullrich A, Waterfield MD, et al (2000) Class II phosphoinositide 3-kinases are downstream targets of activated polypeptide growth factor receptors. Mol Cell Biol 20: 3817–3830. 10805725 10.1128/mcb.20.11.3817-3830.2000 PMC85707

[10] Arcaro A, Khanzada UK, Vanhaesebroeck B, Tetley TD, Waterfield MD, et al (2002) Two distinct phosphoinositide 3-kinases mediate polypeptide growth factor-stimulated PKB activation. Embo J 21: 5097–5108. 12356726 10.1093/emboj/cdf512 PMC129034

[11] Falasca M, Hughes WE, Dominguez V, Sala G, Fostira F, et al (2007) The role of phosphoinositide 3-kinase C2alpha in insulin signaling. J Biol Chem 282: 28226–28236. 17644513 10.1074/jbc.M704357200

[12] Wheeler M, Domin J (2001) Recruitment of the class II phosphoinositide 3-kinase C2beta to the epidermal growth factor receptor: role of Grb2. Mol Cell Biol 21: 6660–6667. 11533253 10.1128/MCB.21.19.6660-6667.2001 PMC99811

[13] Brown RA, Domin J, Arcaro A, Waterfield MD, Shepherd PR (1999) Insulin activates the alpha isoform of class II phosphoinositide 3-kinase. J Biol Chem 274: 14529–14532. 10329640 10.1074/jbc.274.21.14529

[14] Gaidarov I, Smith ME, Domin J, Keen JH (2001) The class II phosphoinositide 3-kinase C2alpha is activated by clathrin and regulates clathrin-mediated membrane trafficking. Mol Cell 7: 443–449. 11239472 10.1016/s1097-2765(01)00191-5

[15] Gaidarov I, Zhao Y, Keen JH (2005) Individual phosphoinositide 3-kinase C2alpha domain activities independently regulate clathrin function. J Biol Chem 280: 40766–40772. 16215232 10.1074/jbc.M507731200

[16] Maffucci T, Cooke FT, Foster FM, Traer CJ, Fry MJ, et al (2005) Class II phosphoinositide 3-kinase defines a novel signaling pathway in cell migration. J Cell Biol 169: 789–799. 15928202 10.1083/jcb.200408005 PMC2171608

[17] Katso RM, Pardo OE, Palamidessi A, Franz CM, Marinov M, et al (2006) Phosphoinositide 3-Kinase C2beta regulates cytoskeletal organization and cell migration via Rac-dependent mechanisms. Mol Biol Cell 17: 3729–3744. 16775008 10.1091/mbc.E05-11-1083 PMC1593155

[18] Domin J, Harper L, Aubyn D, Wheeler M, Florey O, et al (2005) The class II phosphoinositide 3-kinase PI3K-C2beta regulates cell migration by a PtdIns3P dependent mechanism. J Cell Physiol 205: 452–462. 16113997 10.1002/jcp.20478

[19] Ridley AJ, Schwartz MA, Burridge K, Firtel RA, Ginsberg MH, et al (2003) Cell migration: integrating signals from front to back. Science 302: 1704–1709. 14657486 10.1126/science.1092053

[20] Cain RJ, Ridley AJ (2009) Phosphoinositide 3-kinases in cell migration. Biol Cell 101: 13–29. 19055486 10.1042/BC20080079

[21] Velichkova M, Juan J, Kadandale P, Jean S, Ribeiro I, et al (2010) Drosophila Mtm and class II PI3K coregulate a PI(3)P pool with cortical and endolysosomal functions. J Cell Biol 190: 407–425. 20696708 10.1083/jcb.200911020 PMC2922644

[22] Seok YM, Azam MA, Okamoto Y, Sato A, Yoshioka K, et al (2010) Enhanced Ca2+-dependent activation of phosphoinositide 3-kinase class IIalpha isoform-Rho axis in blood vessels of spontaneously hypertensive rats. Hypertension 56: 934–941. 20921425 10.1161/HYPERTENSIONAHA.110.160853

[23] Jaffe AB, Hall A (2005) Rho GTPases: biochemistry and biology. Annu Rev Cell Dev Biol 21: 247–269. 16212495 10.1146/annurev.cellbio.21.020604.150721

[24] Nobes CD, Hall A (1999) Rho GTPases control polarity, protrusion, and adhesion during cell movement. J Cell Biol 144: 1235–1244. 10087266 10.1083/jcb.144.6.1235 PMC2150589

[25] Etienne-Manneville S, Hall A (2001) Integrin-mediated activation of Cdc42 controls cell polarity in migrating astrocytes through PKCzeta. Cell 106: 489–498. 11525734 10.1016/s0092-8674(01)00471-8

[26] Eva A, Aaronson SA (1985) Isolation of a new human oncogene from a diffuse B-cell lymphoma. Nature 316: 273–275. 3875039 10.1038/316273a0

[27] Eva A, Vecchio G, Diamond M, Tronick SR, Ron D, et al (1987) Independently activated dbl oncogenes exhibit similar yet distinct structural alterations. Oncogene 1: 355–360. 3330779

[28] Ron D, Tronick SR, Aaronson SA, Eva A (1988) Molecular cloning and characterization of the human dbl proto-oncogene: evidence that its overexpression is sufficient to transform NIH/3T3 cells. EMBO J 7: 2465–2473. 3056717 10.1002/j.1460-2075.1988.tb03093.x PMC457116

[29] Ron D, Graziani G, Aaronson SA, Eva A (1989) The N-terminal region of proto-dbl down regulates its transforming activity. Oncogene 4: 1067–1072. 2674851

[30] Graziani G, Ron D, Eva A, Srivastava SK (1989) The human dbl-proto-oncogene product is a cytoplasmic phosphoprotein which is associated with the cytoskeletal matrix. Oncogene 4: 823–829. 2666904

[31] Kauppinen KP, Duan F, Wels JI, Manor D (2005) Regulation of the Dbl proto-oncogene by heat shock cognate protein 70 (Hsc70). J Biol Chem 280: 21638–21644. 15802271 10.1074/jbc.M413984200

[32] Kamynina E, Kauppinen K, Duan F, Muakkassa N, Manor D (2007) Regulation of proto-oncogenic dbl by chaperone-controlled, ubiquitin-mediated degradation. Mol Cell Biol 27: 1809–1822. 17178836 10.1128/MCB.01051-06 PMC1820456

[33] Bi F, Debreceni B, Zhu K, Salani B, Eva A, et al (2001) Autoinhibition mechanism of proto-Dbl. Mol Cell Biol 21: 1463–1474. 11238883 10.1128/MCB.21.5.1463-1474.2001 PMC86692

[34] Zheng Y (2001) Dbl family guanine nucleotide exchange factors. Trends Biochem Sci 26: 724–732. 11738596 10.1016/s0968-0004(01)01973-9

[35] Rossman KL, Der CJ, Sondek J (2005) GEF means go: turning on RHO GTPases with guanine nucleotide-exchange factors. Nat Rev Mol Cell Biol 6: 167–180. 15688002 10.1038/nrm1587

[36] Buchsbaum RJ (2007) Rho activation at a glance. J Cell Sci 120: 1149–1152. 17376960 10.1242/jcs.03428

[37] Scita G, Tenca P, Frittoli E, Tocchetti A, Innocenti M, et al (2000) Signaling from Ras to Rac and beyond: not just a matter of GEFs. Embo J 19: 2393–2398. 10835338 10.1093/emboj/19.11.2393 PMC212757

[38] Innocenti M, Frittoli E, Ponzanelli I, Falck JR, Brachmann SM, et al (2003) Phosphoinositide 3-kinase activates Rac by entering in a complex with Eps8, Abi1, and Sos-1. J Cell Biol 160: 17–23. 12515821 10.1083/jcb.200206079 PMC2172734

[39] Posern G, Sotiropoulos A, Treisman R (2002) Mutant actins demonstrate a role for unpolymerized actin in control of transcription by serum response factor. Mol Biol Cell 13: 4167–4178. 12475943 10.1091/mbc.02-05-0068 PMC138624

[40] Das M, Scappini E, Martin NP, Wong KA, Dunn S, et al (2007) Regulation of neuron survival through an intersectin-phosphoinositide 3′-kinase C2beta-AKT pathway. Mol Cell Biol 27: 7906–7917. 17875942 10.1128/MCB.01369-07 PMC2169155

[41] Broome MA, Hunter T (1996) Requirement for c-Src catalytic activity and the SH3 domain in platelet-derived growth factor BB and epidermal growth factor mitogenic signaling. J Biol Chem 271: 16798–16806. 8663329 10.1074/jbc.271.28.16798

[42] Moolenaar WH (1995) Lysophosphatidic acid, a multifunctional phospholipid messenger. J Biol Chem 270: 12949–12952. 7768880 10.1074/jbc.270.22.12949

[43] Hill CS, Wynne J, Treisman R (1995) The Rho family GTPases RhoA, Rac1, and CDC42Hs regulate transcriptional activation by SRF. Cell 81: 1159–1170. 7600583 10.1016/s0092-8674(05)80020-0

[44] Arsenian S, Weinhold B, Oelgeschlager M, Ruther U, Nordheim A (1998) Serum response factor is essential for mesoderm formation during mouse embryogenesis. Embo J 17: 6289–6299. 9799237 10.1093/emboj/17.21.6289 PMC1170954

[45] Miralles F, Posern G, Zaromytidou AI, Treisman R (2003) Actin dynamics control SRF activity by regulation of its coactivator MAL. Cell 113: 329–342. 12732141 10.1016/s0092-8674(03)00278-2

[46] van den Heuvel AP, Schulze A, Burgering BM (2005) Direct control of caveolin-1 expression by FOXO transcription factors. Biochem J 385: 795–802. 15458387 10.1042/BJ20041449 PMC1134756

[47] Burgering BM, Medema RH (2003) Decisions on life and death: FOXO Forkhead transcription factors are in command when PKB/Akt is off duty. J Leukoc Biol 73: 689–701. 12773501 10.1189/jlb.1202629

[48] Engelman JA, Zhang X, Galbiati F, Volonte D, Sotgia F, et al (1998) Molecular genetics of the caveolin gene family: implications for human cancers, diabetes, Alzheimer disease, and muscular dystrophy. Am J Hum Genet 63: 1578–1587. 9837809 10.1086/302172 PMC1377628

[49] Engelman JA, Wykoff CC, Yasuhara S, Song KS, Okamoto T, et al (1997) Recombinant expression of caveolin-1 in oncogenically transformed cells abrogates anchorage-independent growth. J Biol Chem 272: 16374–16381. 9195944 10.1074/jbc.272.26.16374

[50] Galbiati F, Volonte D, Engelman JA, Watanabe G, Burk R, et al (1998) Targeted downregulation of caveolin-1 is sufficient to drive cell transformation and hyperactivate the p42/44 MAP kinase cascade. Embo J 17: 6633–6648. 9822607 10.1093/emboj/17.22.6633 PMC1171009

[51] Frisch SM, Screaton RA (2001) Anoikis mechanisms. Curr Opin Cell Biol 13: 555–562. 11544023 10.1016/s0955-0674(00)00251-9

[52] Whitehead IP, Campbell S, Rossman KL, Der CJ (1997) Dbl family proteins. Biochim Biophys Acta 1332: F1–23. 9061011 10.1016/s0304-419x(96)00040-6

[53] Kato-Stankiewicz J, Ueda S, Kataoka T, Kaziro Y, Satoh T (2001) Epidermal growth factor stimulation of the ACK1/Dbl pathway in a Cdc42 and Grb2-dependent manner. Biochem Biophys Res Commun 284: 470–477. 11394904 10.1006/bbrc.2001.5004

[54] Kato J, Kaziro Y, Satoh T (2000) Activation of the guanine nucleotide exchange factor Dbl following ACK1-dependent tyrosine phosphorylation. Biochem Biophys Res Commun 268: 141–147. 10652228 10.1006/bbrc.2000.2106

[55] Galisteo ML, Yang Y, Urena J, Schlessinger J (2006) Activation of the nonreceptor protein tyrosine kinase Ack by multiple extracellular stimuli. Proc Natl Acad Sci U S A 103: 9796–9801. 16777958 10.1073/pnas.0603714103 PMC1502533

[56] Russo C, Gao Y, Mancini P, Vanni C, Porotto M, et al (2001) Modulation of oncogenic DBL activity by phosphoinositol phosphate binding to pleckstrin homology domain. J Biol Chem 276: 19524–19531. 11278560 10.1074/jbc.M009742200

[57] Hall A, Nobes CD (2000) Rho GTPases: molecular switches that control the organization and dynamics of the actin cytoskeleton. Philos Trans R Soc Lond B Biol Sci 355: 965–970. 11128990 10.1098/rstb.2000.0632 PMC1692798

[58] Jiang K, Sun J, Cheng J, Djeu JY, Wei S, et al (2004) Akt mediates Ras downregulation of RhoB, a suppressor of transformation, invasion, and metastasis. Mol Cell Biol 24: 5565–5576. 15169915 10.1128/MCB.24.12.5565-5576.2004 PMC419878

[59] Coleman ML, Olson MF (2002) Rho GTPase signalling pathways in the morphological changes associated with apoptosis. Cell Death Differ 9: 493–504. 11973608 10.1038/sj.cdd.4400987

[60] Perona R, Esteve P, Jimenez B, Ballestero RP, Ramon y Cajal S, et al (1993) Tumorigenic activity of rho genes from Aplysia californica. Oncogene 8: 1285–1292. 8479750

[61] Qiu RG, Chen J, Kirn D, McCormick F, Symons M (1995) An essential role for Rac in Ras transformation. Nature 374: 457–459. 7700355 10.1038/374457a0

[62] Qiu RG, Chen J, McCormick F, Symons M (1995) A role for Rho in Ras transformation. Proc Natl Acad Sci U S A 92: 11781–11785. 8524848 10.1073/pnas.92.25.11781 PMC40486

[63] Liu Z, Sun C, Zhang Y, Ji Z, Yang G (2010) Phosphatidylinositol 3-kinase-C2beta inhibits cisplatin-mediated apoptosis via the Akt pathway in oesophageal squamous cell carcinoma. J Int Med Res 39: 1319–1332. 10.1177/147323001103900419 21986133

