# Supplementary figures and images for: Phosphoinositide 3-Kinase C2β Regulates RhoA and the Actin Cytoskeleton through an Interaction with Dbl
Source: PLoS One. 2012 Sep 12;7(9):e44945. doi: 10.1371/journal.pone.0044945 (PMC3440356; doi:10.1371/journal.pone.0044945)

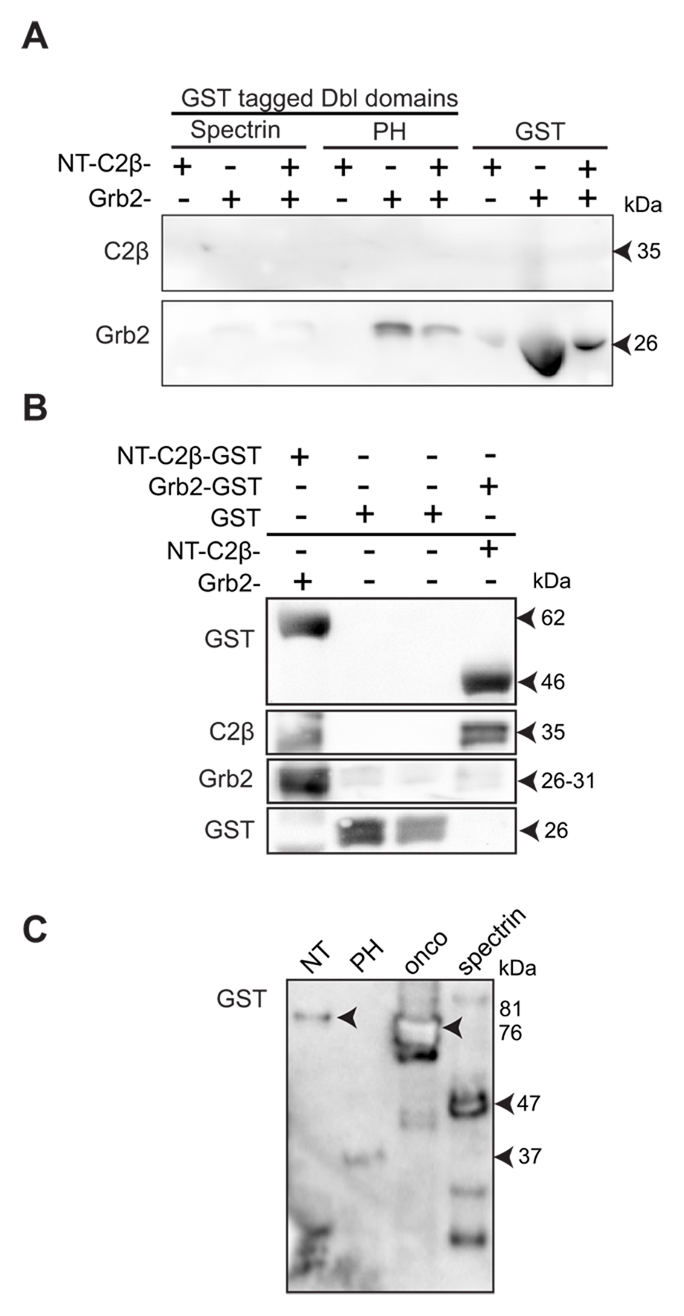

Supplement: Figure S1 — PI3KC2β and Grb2 do not associate directly with Dbl. (A) GST-tagged and gluthatione-sepharose beads conjugated spectrin- and PH- Dbl domains, and GST as control, were incubated in vitro with GST-truncated soluble PI3KC2β N-terminal domain (NT-C2β) (∼35 kDa) and Grb2 (26 kDa). To detect an interaction wih Dbl domains samples were subjected to immunoblotting with indicated antibodies. Experiment was repeated four times with similar results, independently of buffer used for the reaction (Brij96 or Triton 1%). One representative blot is shown (Brij96 1% used for the incubation). (B) Control in vitro reactions, which utilized immobilized on gluthatione-sepharose beads GST-tagged NT-C2β domain and Grb2, and GST as control, were incubated in vitro with GST-truncated soluble NT-C2β domain (∼35 kDa) and Grb2 (26 kDa). Samples were subjected to SDS-PAGE analysis and the interaction between NT-C2β and Grb2 was detected with anti-GST antibody by western blot analysis. (C) Representative immunoblot of Dbl domains separated on SDS-PAGE and analysed by wester blot with anti-GST antibody. (TIF) [file pone.0044945.s001.tif]

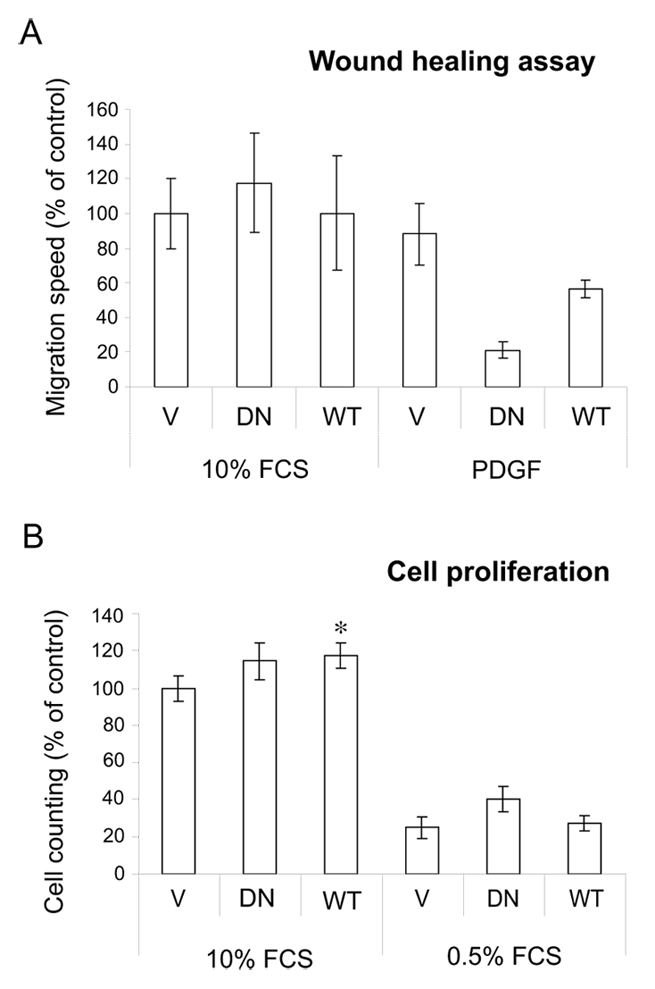

Supplement: Figure S2 — PI3KC2β regulates wound healing properties. (A) Wound healing assay in NIH3T3-V, -C2β-DN and -C2β-WT cells, which were grown in 10% FCS or were serum-starved over night and stimulated with 10 nM PDGF. Migration rate was monitored for 16 h by phase contrast microscopy. Graph presents quantitative analysis of time-lapse microscopy pictures. Data are mean ± SD of two independent experiments. (B) Cell proliferation of NIH3T3-V, -C2β-DN and -C2β-WT cells, which were growing for 48 h either in 10% or 0.5% FCS was performed. The number of viable cells was determined based on Trypan Blue exclusion and cell counting. Data are mean ± SD of three independent experiments. *, p<0.05. (TIF) [file pone.0044945.s002.tif]
